# COVID-19 diagnosis by RT-qPCR in alternative specimens

**DOI:** 10.1590/0074-02760210085

**Published:** 2021-08-13

**Authors:** Cássia Cristina Alves Gonçalves, Shana Priscila Coutinho Barroso, Alice Laschuk Herlinger, Rafael de Mello Galliez, Tailah Bernardo de Almeida, Lidia Theodoro Boullosa, Erica Ramos dos Santos Nascimento, Jessica M de Almeida, Raissa Mirella dos Santos Cunha da Costa, Tatiana Monteiro da Paixão, José Nelson dos Santos Silva Couceiro, Thiago Silva Frauches, Wilson Rodrigues de Souza Jr, Andréa Ribeiro Costa, Débora Souza Faffe, Isabela de Carvalho Leitão, Bianca Ortiz da Silva, Guilherme Sant’Anna de Lira, Isabela Labarba Carvalho de Almeida, Orlando da Costa Ferreira, UFRJ-Covid19 Workgroup, Terezinha Marta Pereira Pinto Castiñeiras, Diana Mariani, Amilcar Tanuri

**Affiliations:** 1 Universidade Federal do Rio de Janeiro, Instituto de Biologia, Departamento de Genética, Laboratório de Virologia Molecular, Rio de Janeiro, RJ, Brasil; 2 Hospital Naval Marcílio Dias, Instituto de Pesquisas Biomédicas, Laboratório de Biologia Molecular, Rio de Janeiro, RJ, Brasil; 3 Universidade Federal do Rio de Janeiro, Faculdade de Medicina, Departamento de Doenças Infecciosas e Parasitárias, Rio de Janeiro, RJ, Brasil; 4 Instituto de Estudos do Mar Almirante Paulo Moreira, Departamento de Biotecnologia Marinha, Arraial do Cabo, RJ, Brasil; 5 Hospital Naval Marcílio Dias, Divisão de Doenças Infecto-Parasitárias, Rio de Janeiro, RJ, Brasil; 6 Universidade Federal do Rio de Janeiro, Instituto de Microbiologia Paulo de Góes, Rio de Janeiro, RJ, Brasil; 7 LACEN - Laboratório Central Dr Francisco Rimolo Neto, Maricá, RJ, Brasil; 8 Universidade Federal do Rio de Janeiro, Instituto de Biofísica, Rio de Janeiro, RJ, Brasil; 9 Universidade Federal do Rio de Janeiro, Centro de Ciências da Saúde, Decania, Rio de Janeiro, RJ, Brasil; 10 Universidade Federal do Rio de Janeiro, Rio de Janeiro, RJ, Brasil

**Keywords:** SARS-CoV-2, diagnosis, saliva, gingival fluid, alternative specimens

## Abstract

**BACKGROUND:**

The high demand for adequate material for the gold standard reverse transcription real-time polymerase chain reaction (RT-qPCR)-based diagnosis imposed by the Coronavirus disease 2019 (COVID-19) pandemic, combined with the inherent contamination risks for healthcare workers during nasopharyngeal swab (NP) sample collection and the discomfort it causes patients, brought the need to identify alternative specimens suitable for the diagnosis of severe acute respiratory syndrome coronavirus 2 (SARS-CoV-2).

**OBJECTIVES:**

The aim of this work was to compare saliva and gingival fluid swabs to NP swabs as specimens for RT-qPCR-based SARS-CoV-2 diagnosis.

**METHODS:**

We compared gingival fluid swabs (n = 158) and saliva (n = 207) to the rayon-tipped NP swabs obtained from mild-symptomatic and asymptomatic subjects as specimens for RT-qPCR for SARS-CoV-2 detection.

**FINDINGS:**

When compared to NP swabs, gingival fluid swabs had a concordance rate of 15.4% among positive samples, zero among inconclusive, and 100% among negative ones. For saliva samples, the concordance rate was 67.6% among positive samples, 42.9% among inconclusive, and 96.8% among negative ones. However, the concordance rate between saliva and NP swabs was higher (96.9%) within samples with lower cycle threshold (Ct) values (Ct > 10 ≤ 25).

**MAIN CONCLUSIONS:**

Our data suggests that whereas gingival fluid swabs are not substitutes for NP swabs, saliva might be considered whenever NP swabs are not available or recommended.

In December 2019, China alerted the world of the detection of a new human coronavirus infection. In March 2020, the World Health Organization (WHO) declared the Coronavirus disease 2019 (COVID-19) outbreak a pandemic, which currently accounts for 173,005,553 cases and has reported 3,727,605 deaths worldwide. In Brazil, the first reported cases date from late February 2020, and, as of June 6, 2021, over 16 million confirmed cases have been reported, resulting in nearly half million deaths (https://covid19.who.int/); and currently, against the world trend, the Brazilian scenario is dramatic as we are facing an unprecedented rise in the number of cases and deaths. To reduce the transmission, scientists all over the world are working on the development of novel diagnostic and treatment methods as well as vaccines for this new disease.^1^ Due to the rapid community transmission of COVID-19, there is consensus among health institutions suggesting that the best strategy to overcome COVID-19 is large-scale testing, enabling a rapid diagnosis, and isolation of infected subjects to block viral transmission.^2^

The gold standard method for severe acute respiratory syndrome coronavirus 2 (SARS-CoV-2) diagnosis is the reverse transcription real-time polymerase chain reaction (RT-qPCR), which is able to detect viral RNA from upper respiratory specimens, such as oropharyngeal or nasopharyngeal (NP) swabs, or lower respiratory ones, such as endotracheal aspirate or bronchoalveolar lavage;^3^ ^,^ ^4^ specifically the synthetic fiber-tipped swabs (rayon) which are recognised by both the WHO and the Centre for Disease Control and Prevention (CDC) for this purpose.^5^ The collection of oropharyngeal and NP swabs is invasive, uncomfortable for patients, causing cough and aerosol of viruses and leading to increased risk of viral transmission due to the close contact between patients and healthcare workers during the collection procedure.^6^ ^,^ ^7^ Therefore, biosafety associated to the collection of such specimens poses yet another challenge of COVID-19 diagnosis.

The presence of infectious SARS-CoV-2 in saliva, as well as the expression of SARS-CoV-2 entry factors ACE2 and TMPRSS members in epithelial cells of glands and oral mucosa have already been demonstrated.^8^ Therefore, oral cavity samples, such as saliva and gingival swabs, come forth as alternative specimens for SARS-CoV-2 detection.

The aim of this study was to evaluate alternative specimens for RT-qPCR-based COVID-19 diagnosis. With an attempt to simplify the sample collection procedure in order to both make it less invasive and uncomfortable for patients, while more secure for the healthcare workers. Therefore, we compared the use of gingival fluid swabs and saliva samples with the gold-standard rayon-tipped NP swab as speciments for SARS-CoV-2 detection by RT-qPCR.

## SUBJECTS AND METHODS

*Patient recruitment* - A total of 364 individuals who sought clinical care at Marcílio Dias Naval Hospital (HNMD), at the COVID-19 Diagnostic Centre of the Federal University of Rio de Janeiro, and at COVID-19 diagnosis tents in the Maricá municipality (Rio de Janeiro State) were included in the study. These individuals either presented flu-like symptoms (such as anosmia, cough and fever, among others), and thus were considered as suspects for acute COVID-19, or had close contact with infected subjects, regardless of symptoms. Thus, the present study only included mild-symptomatic and asymptomatic subjects. Among them, 157 were included in the gingival fluid analysis, and 207 in the saliva sample analysis. Only individuals aged ≥ 18 years of age were included in the study.

Sample Collection

*NP swabs* - NP swab collection was performed by introducing a rayon-tipped swab into the nasal cavity, directing it upwards (towards the eyes), at an angle of 30º to 45º in relation to the upper lip. The swab was rotated 10 times while in the NP cavity, and allowed to rest for 30 seconds prior to removal. Then, it was placed in a tube containing 2 mL of viral transport medium (VTM).

*Gingival fluid* - The oral fluid from the gingiva (hereafter referred to as gingival fluid) was collected on the same day as the NP swab. A rayon-tipped swab was rubbed against both the maxillary and the mandibular gingiva. After rubbing both right and left sides, the swab was placed in a tube with 2 mL of VTM.

*Saliva* - Saliva was collected on the same day as the NP swabs. Patients were asked to spit in a sterile recipient, producing at least 1 mL of saliva. Samples were kept refrigerated until further analysis.

*RNA extraction and RT-qPCR* - Total viral RNA from swabs and saliva were extracted in a Maxwell^®^ 16 Instrument (Promega, WI, USA), using the Maxwell^®^16 Viral Total Nucleic Acid Purification Kit (Promega), according to manufacturer’s instructions. Viral RNA was detected using the SARS-CoV-2 (2019-nCoV) CDC qPCR Probe Assay (Integrated DNA Technologies, IA, USA) targeting the SARS-CoV-2 N1 and N2 genes, and the human ribonuclease P (RNaseP) gene (endogenous control), and the GoTaq^®^ Probe 1-Step RT-qPCR System (Promega), according to the manufacturer’s instructions. All reactions were paired and performed in a Rotor-Gene Q Thermocycler (Qiagen, Hilden, Germany) or in a 7500 Thermal Cycler (Applied Biosystems, CA, USA).

RT-qPCR results were interpreted as follows: Positive for SARS-CoV-2: both targets (N1 and N2) amplified with cycle threshold (Ct) ≤ 37; inconclusive: only one target amplified with Ct ≤ 37, or both targets amplified with Ct > 37 ≤ 40; negative: one target amplified with Ct > 37 ≤ 40, both targets amplified with Ct > 40, or absence of amplification.

*Statistical analysis* - The Kolmogorov-Smirnov test was used to test the normality of data. Two-tailed T test was performed to compare data under a Gaussian distribution, whereas two-tailed Wilcoxon matched pairs test was used for non-normally distributed data. All analyses were performed using Graph PadPrism v.5, with a p ≤ 0.05 considered statistically significant. Data are shown as mean ± standard deviation (SD), and the 95% confidence interval (CI) is informed.

*Ethics* - This study was approved by the Institutional Review Boards from Marcílio Dias Naval Hosptial (HNMD) (protocol number 32382820.3.0000.5256), and by the National Commission for Research Ethics (CONEP, Brazil; protocol #30161620.0.0000.5257; approval #3953368). We obtained written informed consent for all individuals included in this study.

## RESULTS

*Biographic data* - Samples were obtained from 364 individuals from both sexes. NP swab samples (taken from all individuals) were compared with gingival fluid samples from 158 individuals and with saliva samples from the other 207 individuals. Regarding individuals in the gingival fluid cohort, 108 (68.79%) were female. The mean age was 39 ±13 years of age (range: 18- 80 years of age). The average number of days from the onset of symptoms to sample collection was 4.71 ± 2.37 (range: 1-15 days) and six subjects were asymptomatic (3.82%).

As for patients participating in the saliva sample cohort, 98 were female (47.57%). The mean age was 43 ± 15.56 years of age (range: 18 - 89 years of age). The average number of days from the onset of symptoms to sample collection was 7.11 ± 11.39 (range: 1-110 days); however, this data was not informed for 43 subjects (20.77%), and 38 subjects were asymptomatic (18.36%).

*Comparison between RT-qPCR results* - In regards to the gingival swab group, according to the NP swab results, 26 (16.56%) subjects were positive, one (0.64%) was inconclusive, and 130 (82.80%) were negative. When comparing gingival swab results the concordance rate was 15.38% (4/26) among positive NP results (with two (7.69%) inconclusive and 20 (76.92%) negative gingival swab results), zero among the inconclusive NP sample (which was negative in the gingival fluid), and 100% among negative NP swab samples [Figure (A) and Supplementary data (Table I)].

The Ct values obtained from gingival swabs were, on average, over 10 Cts higher than NP for both N1 (p = 0.00313) and N2 (p = 0.0010). The endogenous RP control was also higher among gingival samples, but the average difference was two Cts (p < 0.0001) [Supplementary data (Table II)].

**Figure:**
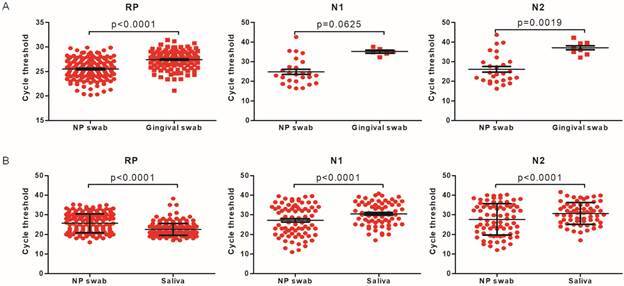
Comparative reverse transcription real-time polymerase chain reaction (RT-qPCR) results from (A) gingival fluid swab and nasopharyngeal (NP) swab specimens, and (B) saliva and NP swab specimens. Data is shown as mean ± standard deviation (SD) of cycle threshold (Ct) values for severe acute respiratory syndrome coronavirus 2 (SARS-CoV-2) N1 and N2 genes, and the endogenous control RNase P (RP).

In the saliva group, NP swab specimens yielded 71 (34.29%) positive, 7 (3.38%) inconclusive, and 129 (63.32%) negative results. When compared to NP swab samples, there was a concordance rate of 67.61% among positive, 42.86% among inconclusive, and 96.83% among the negative results [Figure (B) and Supplementary data (Table III)]. Whereas the Ct values for N1 and N2 were 3 cycles higher on average among saliva samples (p < 0.0001), the mean RT Ct was lower among saliva samples when compared to NP swabs (p < 0.0001) [Supplementary data (Table IV)]. When the results were stratified according to N1/N2 Ct values for NP swab samples, a better concordance was observed. Among samples with N1 or N2 Ct > 10 ≤ 25 (n = 32) the concordance rate was 96.88% (n = 31/32), in which only one sample which was positive in the NP swab was inconclusive in saliva. When N1 and N2 Ct values were > 25 ≤ 30 (n = 10) the concordance rate was 70% (n = 7/10), in which two positive samples in the NP swab were negative, and one was inconclusive when saliva was used. Finally, when N1/N2 CT values were > 30 (n = 156) the concordance rate was 14.29% (n = 3/21) among positive, 33.33% (n = 2/6) among inconclusive, and 94.57% (n = 122/129) among negative results.

## DISCUSSION

In an effort to validate alternative sample types that would be easier, less uncomfortable and safer to be collected, we evaluated gingival swabs and saliva in comparison to NP swabs as specimens for COVID-19 diagnosis by RT-qPCR, in mild-symptomatic and asymptomatic individuals. Our rationale for selecting these sample types, besides from the practical aspect, is the increasing evidence that SARS-CoV-2 could be shed in the oral cavity. In this regard, the spread of SARS-CoV-2 through speech-release saliva droplets has already been demonstrated experimentally,^9^ and WHO has acknowledged SARS-CoV-2 spreads primarily through saliva droplets or discharges from the nose.^10^ Moreover, ACE2 (angiotensin-converting enzyme 2) expression has already been detected in salivary glands,^11^ suggesting this epithelium is permissive to SARS-CoV-2 infection.

Considering that oropharyngeal swabs might be used for SARS-CoV-2, a few studies have explored the applicability of buccal swab specimens for COVID-19 diagnosis. The first study compared NP swab and buccal swabs from 11 symptomatic or asymptomatic hospitalised SARS-CoV-2 infected children.^12^ In this study both NP and buccal swabs were collected daily until NP swab RT-qPCR was negative for two consecutive days, with SARS-CoV-2 being detected in at least one buccal collection in 81.8% of cases. The second study included 42 individual who tested positive for SARS-CoV-2 by NP swab RT-qPCR within seven days prior to self-collected buccal swab (both cheeks, above and below the tongue, both gums, and on the hard palate), and a positive agreement of 56.7% was observed.^13^ Herein we tested gingival fluid as a specimen for SARS-CoV-2 detection by RT-qPCR, and we found a poor concordance rate (15.38%) among RT-qPCR positive samples from NP swabs. Although our data is in agreement with previous data, our concordance rate among positive results was lower than the those reported by Ku et al.^14^ and Kam et al.^12^. This variation may be due to the study designs that differed regarding the moment of sample collection in relation to NP swab collection, the number of sample collection time points (single or multiple days), and the way (self- or healthcare professional-performed collection) samples were collected. Nonetheless all three studies conclude that swabs obtained from the oral cavity are not suitable for accurate COVID-19 diagnosis by RT-qPCR.

As for saliva, it has already been shown to be a possible reliable specimen for SARS-CoV-2 detection and this sample type has been previously used for the detection of other respiratory viruses, including SARS-CoV.^14^

The main argument behind the use of saliva as a specimen for SARS-CoV-2 detection, apart from the comfort of the patient during sample collection, was biosafety. Therefore, in order to simulate a self-collecting sampling in our cohort, we did not assist every individual collection, although every subject was advised on how to perform the collection. By doing so, we obtained a concordance rate among NP swab positive samples of 67.61%. It has already been proposed that the diagnostic value of saliva for SARS-CoV-2 detection seems to depend on how the specimen has been collected, with saliva from deep throat being more appropriate for early COVID-19 diagnosis.^15^ Therefore, some inconsistencies observed when comparing results from NP swabs and saliva may be due to the amount and type of saliva produced.

On the other hand, when analysing Ct values for the endogenous control (RP), we found that saliva yields lower values when compared to NP swab samples. This finding suggests that sample collection was performed successfully, and that the failure to detect SARS-CoV-2 RNA was due to viral load. This idea is corroborated by the findings among samples with Ct values ≤ 25, which showed a concordance rate of 96.88% as opposed to 66.7% when raw data was used. However, when data was stratified by days since symptom onset, no improvement in the concordance rate between paired NP swab and saliva samples was observed (data not shown). This indicates the moment of sample collection does not affect the applicability of saliva as a sample for SARS-CoV-2 RT-qPCR-based detection, when compared to the NP swab samples.

However, stratification according to days since symptom onset might not be the best parameter to adjust the analysis, since it has already been shown that detection of SARS-CoV-2 in asymptomatic individuals was more sensitive in saliva samples when compared to matched NP swab samples.^16^ On the other hand, in a different study with hospitalised patients, authors have described a concordance rate between saliva and NP swab samples of 91.67%, with viral load in saliva decreasing over time.^7^ Conversely, a third study, which evaluated SARS-CoV-2 detection in samples collected at different time points, has shown that the majority of patients had unaltered results (similar Ct values) over a 4-day interval; however two individuals had positive RT-qPCR using saliva samples after NP swab samples were negative.^17^ Finally, a fourth study has shown that although the average NP Ct value was lower than that of saliva specimens (which is consistent with our findings), these differences were only statistically significant within the first four days from symptom onset.^18^ Therefore, although it is still not clear whether SARS-CoV-2 loads in saliva vary according to disease course or viral shedding in this specimen, all available data so far - including ours - seems to agree on the diagnostic value of saliva for COVID-19 diagnosis, with clear benefits towards patient comfort during sample collection and decreased exposure of healthcare workers, avoiding iatrogenic transmission.^7^ ^,^ ^15^ Moreover, saliva as a specimen for COVID-19 RT-qPCR-based diagnosis should be considered whenever swabs are unavailable or its use is not recommended.

The data presented herein confirm previous data advising against the use of buccal/gingival fluids for RT-qPCR SARS-CoV-2 detection, we bring further evidence that saliva may be considered to this aim. Despite SARS-CoV-2 detection in saliva not being as sensitive as NP swabs, practical issues regarding patient comfort, availability of trained personnel to perform sample collection, and exposure of these personnel to SARS-CoV-2 aerosols should be evaluated when considering saliva for COVID-19 diagnosis.
