# Supplementary material for: COVID-19 diagnosis by RT-qPCR in alternative specimens
Source: Mem Inst Oswaldo Cruz. 2021 Aug 13;116:e210085. doi: 10.1590/0074-02760210085 (PMC8370469; doi:10.1590/0074-02760210085)
Supplement: Supplementary file 1 [file 1678-8060-mioc-116-e210085-s.pdf]

TABLE I

Concordance between gingival fluid swab and nasopharyngeal swab (NP) specimens in reverse transcription real-time polymerase chain reaction (RT-qPCR)-based Coronavirus disease 2019 (COVID-19) diagnosis

RT-qPCR result	Gingival swab positive (agreement %)	Gingival swab inconclusive (agreement %)	Gingival swab negative (agreement %)	Total (%)
NP swab positive	4 (15.38)	2	20	26 (16.56)
NP swab inconclusive	0	0 (0)	1	1 (0.64)
NP swab negative	0	0	130 (100)	130 (82.8)
Total	4	2	151	157

TABLE II

N1, N2 and reverse transcription real-time polymerase chain reaction (RT-qPCR) cycle threshold (Ct) values for gingival fluid swab and nasopharyngeal swab (NP) specimens

	N1[a]		N2[b]		RP[c]	
Specimen	Mean CT (SD)	95% CI	Mean Ct (SD)	95% CI	Mean Ct (SD)	95% CI
NP swab	24.85 ± 6.482	22.33-27.36	26.30 ± 7.147	23.58-29.02	25.45(2.097)	25.15-25.78
Gingival swab	36.20 ± 3.20	33.24-39.16	37.18 ± 3.032	35.01-39.35	27.36(1.924)	27.03-27.70

a: Wilcoxon matched pairs test $p = 0.0313$; *b*: two-tailed paired T test $p = 0.0010$; *c*: Wilcoxon matched pairs test $p < 0.0001$. SD: standard deviation; CI: confidence interval; RP: RNase P.

TABLE III

Concordance between saliva and nasopharyngeal swab (NP) specimens in reverse transcription real-time polymerase chain reaction (RT-qPCR)-based Coronavirus disease 2019 (COVID-19) diagnosis

RT-qPCR result	Saliva positive (agreement %)	Saliva inconclusive (agreement %)	Saliva negative (agreement %)	Total (%)
NP swab positive	48 (67.61)	3	20	71 (34.29)
NP swab inconclusive	1	3 (42.86)	3	7 (3.38)
NP swab negative	4	3	122 (96.83)	129 (62.32)
Total	52	9	145	207

TABLE IV

N1, N2 and reverse transcription real-time polymerase chain reaction (RT-qPCR) cycle threshold (Ct) values for gingival fluid swab and nasopharyngeal swab (NP) specimens

	N1*		N2*		RP*	
Specimen	Mean CT (SD)	95% CI	Mean Ct (SD)	95% CI	Mean Ct (SD)	95% CI
NP swab	27.34 (7.797)	25.58-29.09	27.35 (8.004)	25.81-29.49	26.68 (4.803)	25.01-26.35
Saliva	30.50 (5.880)	29.08-31.32	30.67 (5.547)	29.26-32.08	22.56 (3.008)	22.15-22.98

**: Wilcoxon matched pairs test $p < 0.0001$; SD: standard deviation; CI: confidence interval; RP: RNase P.

CC BY

*UFRJ-Covid19 Workgroup members

Aliny S Carvalho	Juliana C de Menezes
Anna CP Castiñeiras	Juliana TS Fortuna
Átila D Rossi	Karen S Pereira
Bruno Clarkson	Karyne F Monteiro
Bruno E Dematté	Lendel C da Costa
Camila A Velozo	Kissyla HDP França
Camila Nacif	Laura Z Renault
Camille Victória	Letícia A Corrêa
Leal C de Silva	Liane J Ribeiro
Carolina M Voloch	Liliane TF Cavalcante
Caroline M Nascimento	Luana S Costa
Carolyne LAL da Graça	Lucas M Millioni
Cíntia Policarpo	Luciana J da Costa
Cynthia C Cardoso	Luisa M Higa
Ekaterini S Goudouris	Manoel I do Nascimento
Elaine S da Costa	Marcelo A de Souza
Elisangela C da Silva	Marcelo CP Tôrres
Fábio L Monteiro	Marcela S Durães
Fabio HC Medeiros	Mariana F Campos
Fernanda L dos Santos	Mariane T de Menezes
Fernando L de Castro	Marisa S Correia
Filipe RR Moreira	Mateus R de Queiroz
Francine B Schiffler	Matheus AC Cosentino
Gabriela B Kraychete	Mayla GM de Melo
Gabriele S da Cunha	Mirela D'arc
Gisely NB da Cunha	Pedro HC da Paz
Gustavo PD da Silva	Raquel F Coelho
Harrison J Westgarth	Richard A Maia
Helena A de Oliveira	Rodrigo M Brindeiro
Helena K Toma	Romina C Ferreira
Helena T Scheid	Sérgio M Lisboa
Huang L Fang	Thamiris S Miranda
Inês C Gonçalves	Victor A Ota
Ingrid C da Silva	Victoria C Bastos
Joissy A de Oliveira	Viviane G Gomes
